# Electrospun Nanofibers with Pomegranate Peel Extract as a New Concept for Treating Oral Infections

**DOI:** 10.3390/ma17112558

**Published:** 2024-05-26

**Authors:** Magdalena Paczkowska-Walendowska, Miłosz Ignacyk, Andrzej Miklaszewski, Tomasz Plech, Tomasz M. Karpiński, Jakub Kwiatek, Ewelina Swora-Cwynar, Michał Walendowski, Judyta Cielecka-Piontek

**Affiliations:** 1 Department of Pharmacognosy and Biomaterials, Poznan University of Medical Sciences, Rokietnicka 3, 60-806 Poznan, Poland; m.ignacyk99@gmail.com (M.I.); jpiontek@ump.edu.pl (J.C.-P.); 2 Faculty of Materials Engineering and Technical Physics, Institute of Materials Science and Engineering, Poznan University of Technology, 60-965 Poznan, Poland; andrzej.miklaszewski@put.poznan.pl; 3 Department of Pharmacology, Medical University of Lublin, Radziwillowska 11, 20-080 Lublin, Poland; tomasz.plech@umlub.pl; 4 Department of Medical Microbiology, Medical Faculty, Poznan University of Medical Sciences, Rokietnicka 10, 60-806 Poznan, Poland; tkarpin@ump.edu.pl; 5 Kwiatek Dental Clinic Sp. z o.o., Kordeckiego 22, 60-144 Poznan, Poland; jakubkwiatek@klinikakwiatek.pl; 6 Department of Pharmacology and Phytochemistry, Institute of Natural Fibres and Medicinal Plants—National Research Institute, Wojska Polskiego 71b, 60-630 Poznan, Poland; eswora@ump.edu.pl; 7 Science-Bridge Sp. z o.o., Chociszewskiego 24/8, 60-258 Poznan, Poland; michalwalendowskipoczta@gmail.com

**Keywords:** pomegranate peel, antibacterial, anti-inflammatory, wound healing, oral infections, nanofibers

## Abstract

Pomegranate peel extract is known for its potent antibacterial, antiviral, antioxidant, anti-inflammatory, wound healing, and probiotic properties, leading to its use in treating oral infections. In the first stage of this work, for the first time, using the Design of Experiment (DoE) approach, pomegranate peel extract (70% methanol, temperature 70 °C, and three cycles per 90 min) was optimized and obtained, which showed optimal antioxidant and anti-inflammatory properties. The optimized extract showed antibacterial activity against oral pathogenic bacteria. The second part of this study focused on optimizing an electrospinning process for a combination of polycaprolactone (PCL) and polyvinylpyrrolidone (PVP) nanofibers loaded with the optimized pomegranate peel extract. The characterization of the nanofibers was confirmed by using SEM pictures, XRPD diffractograms, and IR-ATR spectra. The composition of the nanofibers can control the release; in the case of PVP–based nanofibers, immediate release was achieved within 30 min, while in the case of PCL/PVP, controlled release was completed within 24 h. Analysis of the effect of different scaffold compositions of the obtained electrofibers showed that those based on PCL/PVP had better wound healing potential. The proposed strategy to produce electrospun nanofibers with pomegranate peel extract is the first and innovative approach to better use the synergy of biological action of active compounds present in extracts in a patient-friendly pharmaceutical form, beneficial for treating oral infections.

## 1. Introduction

The WHO Global Oral Health Status Report (2022) estimated that severe oral and periodontal disorders affect close to 3.5 billion people worldwide [1], causing tooth problems such as tooth loss, but also systemic diseases such as cardiovascular disease or diabetes [2].

Because local drug delivery methods control drug release, they offer better efficacy and fewer side effects when used to treat periodontitis. An effective treatment plan for periodontitis is based on selecting a suitable bioactive agent, material base, and delivery route [3].

Plant materials have been successfully and extensively employed in the treatment of numerous illnesses, including periodontitis. Plant-based remedies for periodontitis have antimicrobial, anti-inflammatory, and antioxidant properties that also impact the structure of the periodontium [4]. Numerous studies demonstrate the advantages of phytotherapy in the treatment of periodontal diseases and indicate the usefulness of Baikal skullcap roots [5,6], tea leaves [7], or resveratrol-rich plants [8]. There is still little information about the use of pomegranate peel.

Although pomegranate peel (*Punica granatum* L.) makes up half of all fruits, it was previously considered waste [9]. According to recent research, plant material is a valuable waste with potentially beneficial pharmacological effects since it is a rich source of phenols (such as ellagitannins, flavonoids, and anthocyanins), polysaccharides, and its biotransformed metabolites, such as urolithin [10]. Due to its unique phytochemical components, the raw material exhibits numerous health benefits such as antioxidant, anti-inflammatory [11], and antimicrobial activities, as well as those responsible for oral infections and caries [12].

Many innovative techniques are being used to produce new forms in the oral cavity [13,14], one of which is electrospinning [15], a green manufacturing method with no negative impact on the environment and health [16]. The development of nanofibers has made it easier to create and use biomaterials as three-dimensional artificial scaffolds. Since many natural and synthetic biomaterials can easily imitate natural human tissue at the nanoscale, they can be employed as resources for restorative dentistry [17]. A high surface-area-to-volume ratio and nanofibers’ microporous architectures provide an advantage in restoring complex anatomical dental features. When it comes to cell adhesion, proliferation, and differentiation, nanofibers have a lot to offer. They can be employed in dental implants and drug delivery systems within the oral cavity [18,19].

To produce an effective system, it is crucial to use appropriate materials as the basis of the product. One biodegradable synthetic polymer, polycaprolactone (PCL), has gained attention as a class of biomaterials with potential applications in surgery, including sutures, medication administration, devices for internal stabilization of bone fractures, and scaffolds for tissue or organ regeneration [20]. Despite PCL’s good biocompatibility and efficacy in vitro and in vivo, its slow breakdown kinetics and extremely hydrophobic nature may make it unsuitable for use in several biomedical applications where higher absorption rates are required [21]. One approach to overcome this limitation is to add water-soluble, biocompatible polymers to the electrospinning process [22]. This method has also been effectively used to improve the topological, structural, and chemical characteristics of various polymeric meshes to boost their hydrophilicity and biocompatibility. One of the water-soluble polymers is poly(N-vinyl-2-pyrrolidone) (PVP). PVP has excellent biocompatibility, extraordinary water solubility, and the ability to interact with various hydrophilic and hydrophobic materials [23].

Preparing an extract from the plant material with the best properties is necessary to produce an effective pharmaceutical form. Only two works show the process of optimizing the pomegranate peel extraction but do not consider the biological effect [24,25]. Therefore, the first part of the study aimed to optimize the extraction process using ultrasonic-assisted extraction (UAE). The optimized extract can be used to prepare the finished product, and the existing literature data indicate the possibility of using electrospinning to produce packaging based on pomegranate peel [26,27] but do not indicate the possibility of preparing an innovative form of delivery for dental use. Thus, the second part of this work aims to evaluate the likelihood of simultaneous electrospinning of PCL with water-soluble PVP, which will provide important information on effectively tuning the biodegradation rate of electrospun PCL scaffolds but is not limited to the extension of other high-value biocompatible polymers for future biomedical applications, in particular dental, ranging from controlled drug delivery to tissue regeneration.

## 2. Materials and Methods

### 2.1. Plant Material

The plant material used is pomegranate peel (*Punica granatum*), the country of origin is India, and it was purchased from the *NANGA Przemysław Figura* (Blękwit, Poland), Lot No. 33192.

### 2.2. Chemicals

Kaempferol (Phyproof^®^ Reference Substance) as well as rutin (≥95%) were obtained from Sigma-Aldrich (Poznan, Poland). Polyvinylpyrrolidone K30 (PVP) was obtained from BASF (Warsaw, Poland), while polycaprolactone (PCL) average M_n_ 80,000 from Sigma Aldrich (Poznan, Poland). Folin-Ciocalteu reagent, 2,2-Diphenyl-1-picrylhydrazyl (DPPH), gallic acid, neocuprine, 2,4,6-Tris(2-pyridyl)-s-triazine (TPTZ), ferric chloride (III), bovine serum, hyaluronic acid, hyaluronidase, polycaprolactone (PCL), hexadecyltrimethylammonium bromide (CTAB), and bovine gastric mucin were obtained from Sigma-Aldrich (Poznan, Poland). Methanol p. a., cupric chloride (II), ethanol 96%, ammonium acetate p. a., sodium hydroxide and sodium chloride were obtained from Avantor Performance Materials Poland (Gliwice, Poland). Sodium carbonate p. a. was obtained from POCH Gliwice (Gliwice, Poland). Analytical balance hydrochloric acid 0.1 mol/L (0.1 N) was obtained from Chempur (Piekary Śląskie, Poland). High-quality pure water was prepared using a Direct-Q 3 UV Merck Millipore purification system (Burlington, MA, USA).

### 2.3. Pomegranate Peel Extraction Using Design of Experiments

A factor experiment plan was created for three independent variables with three levels of values assigned using the Design of Experiments (DoE) approach (3^2^ complete factorial design) (Statistica 13.3 software, TIBCO Software Inc., Palo Alto, CA, USA). The methanol concentration in extraction mixture, temperature, and time were chosen as independent variables (Table 1).

To prepare each extract, 5.0 g of dried and pulverized pomegranate peel was extracted with a mixture of 50.0 mL of methanol and distilled water in Erlenmeyer flasks. Extraction was assisted by ultrasound (UAE) using an ultrasound bath due to their positive influence on this type of process. The obtained extracts were separated from the plant material by under-pressure filtration as the next step. The extraction process was repeated three times, each time using a fresh portion of the extraction mixture. The three obtained extracts were combined and condensed to achieve a final extract volume of 25.0 mL. The resulting products were stored within a temperature range of 2–8 °C. The final concentration of the extract is 200.0 mg of plant material per milliliter.

### 2.4. Determination of Selected Active Component Content and Total Phenolic Content (TPC)

#### Total Phenolic Content

The contents of active polyphenolic compounds (flavan-3-ols: rutin and kaempferol) were determined using the HPLC method described previously [28].

To assess the Total Polyphenolic Content (TPC) of prepared extracts, the method described by Kikowska et al. with further modifications was employed [29].

### 2.5. Studying the Biological Activity of Pomegranate Peel Extract

#### 2.5.1. Antioxidant Activity

A variety of radical cation-based assays, including 2,2-Diphenyl-1-picrylhydrazyl (DPPH) assay, as well as cupric ion reducing antioxidant capacity (CUPRAC) assay, and ferric ion reducing antioxidant parameter (FRAP) assay, were used to measure antioxidant activity with methods previously published [29].

#### 2.5.2. Anti-Inflammatory Activity

The procedure of hyaluronidase inhibition was determined by the turbidimetric method described previously [29].

### 2.6. Prediction of the DoE Model and Preparation of an Optimized Extract

Based on the experimental studies described above, the DoE model was predicted, and an optimized extract was prepared using the method described above (70% methanol, 70 °C, and 90 min). The extract was subjected to a lyophilization process (a condensation temperature set at −48 °C under reduced pressure (1.030 mbar) for 48 h; CHRIST 1-4 LSC, Osterode am Harz, Germany) to obtain a dry extract, which was used for further research.

#### 2.6.1. The Antioxidative and Anti-Inflammatory Assays

The antioxidative and anti-inflammatory assays were conducted following the procedure outlined in Section 2.5.

#### 2.6.2. Microbiological Activity

The minimal inhibitory concentrations (MIC) of the pomegranate extract were determined using the microdilution method with 96-well plates (Nest Scientific Biotechnology, Wuxi, China). The experimental procedures followed the methodology presented in our previous publications [30,31]. The activity of the pomegranate extract against clinical strains of the yeast *Candida albicans*, clinical strains of the bacteria responsible for wound infections *Staphylococcus aureus* and *Pseudomonas aeruginosa*, the cariogenic bacterium *Streptococcus mutans* ATCC 25175, and the periopathogens *Schaalia odontolytica* (*Actinomyces odontolyticus*) ATCC 17929, *Fusobacterium nucleatum* ATCC 25586, *Porphyromonas gingivalis* ATCC 33277, and *Prevotella intermedia* ATCC 25611 was investigated. Yeasts were cultured on Sabouraud broth, bacteria *S. aureus*, *S. mutans*, and *P. aeruginosa* on tryptone soy broth (TSB), and periopathogens on Schaedler broth (Graso Biotech, Owidz, Poland). Serial dilutions of the extract were performed in the appropriate medium to achieve final concentrations of 50, 25, 12.5, 6.25, and 3.1 mg/mL in the wells. The inoculums were adjusted to obtain a final concentration of 10^5^ CFU/mL for bacteria and 10^4^ CFU/mL for fungi. The plates were then incubated at 37 °C for 24–48 h, and MIC values were determined through visual analysis. To improve reading, color reactions were used: for aerobic pathogens, 2,3,5-triphenyltetrazolium chloride (TTC) (Sigma, Poznań, Poland) and for anaerobic periopathogens, resazurin (Merck, Warsaw, Poland).

### 2.7. Preparation of the Electrospun Nanofibers with Optimized Pomegranate Peel Extract

To produce nanofibers, 6 solutions of polymers/compositions of polymers and extract were prepared (Table 2). Ingredients were dissolved using a mixture of methanol and dichloromethane in a ratio of 1:3 (*v*:*v*).

The electrospinning process was conducted using the NS + Nano Spinner Plus Electrospinning Equipment (Inovenso Ltd., Istanbul, Turkey). The prepared mixtures were loaded into syringes fitted with a metal needle. The distance between the needle and the collector was maintained at 12.0 cm. The flow rate was set to 2.0 mL/min, and the rotation speed of the collector was set to 200 rpm. The efficiency of nanofiber production was determined by comparing the weight of the produced nanofibers with the weight of the substrates. The experiments were conducted at room temperature (about 25 °C), and the humidity did not exceed 40%.

### 2.8. Identification of Obtained Electrospun Nanofibers

#### 2.8.1. Scanning Electron Microscopy (SEM)

SEM images were performed using a scanning electron microscope (Quanta 250 FEG, FEI, Waltham, MA, USA) to assess the morphology of prepared nanofibers. Before analysis, the nanofibers were sputter-coated with gold palladium. The diameters of nanofibers were measured using SEM pictures using the ImageJ program (https://imagej.net/nih-image/, accessed on 25 May 2024).

#### 2.8.2. X-ray Powder Diffraction (XRPD)

The obtained nanofibers were evaluated in a crystallographic assay using X-ray diffraction (XRD) equipment (Panalytical Empyrean, Almelo, The Netherlands) with a copper anode (CuKα—1.54 Å). The measurements were conducted in Bragg–Brentano reflection mode configuration with parameters set to 45 kV and 40 mA. The measurement range was defined from 3° to 60°, with a step size of 0.05° and a measurement time of 45 s per step.

#### 2.8.3. Infrared Spectroscopy with Attenuated Total Reflectance (IR-ATR)

The IR-ATR analysis was conducted in the range of 400 to 4000 cm^−1^ in absorbance mode, with a resolution of 1 cm^−1^. The spectrogram was obtained using an IRTracer-100 spectrophotometer manufactured by Shimadzu (Kyoto, Japan), and operated with LabSolutions IR software (version 1.86 SP2). The spectrophotometer was equipped with a QATR-10 single bounce diamond extended range accessory.

### 2.9. Analysis of Electrospun Nanofibers’ Functionality

#### 2.9.1. Dissolution Studies

Using an Agilent 708-DS dissolution device, electrospun nanofibers were studied for their dissolution behaviors. A typical basket method was applied, stirring at 50 rpm and 37 ± 0.5 °C. Nanofibers were added to 300 mL of artificial saliva solution and adjusted to pH to 6.8 with 1 M HCl. At certain intervals, liquid samples were taken, and an equivalent volume of medium that had been adjusted for temperature was added. A nylon membrane filter with a diameter of 0.45 μm was used to filter the samples. The quantities of rutin in the acceptor solutions were ascertained using the HPLC technique previously mentioned. The studies maintained sink conditions. There were six iterations of the study.

To investigate the release kinetics, the resulting active compound release patterns were fitted to the zero-order, first-order, Higuchi, and Korsmeyer–Peppas models [32].

#### 2.9.2. Mucoadhesive Properties

Mucoadhesive properties were evaluated by measuring the bonding strength between the utilized polymers (PVP, PCL) within nanofibers and mucin. The study followed the procedure outlined by Hassan and Gallo with some modifications [33], employing the viscosimetric technique.

### 2.10. Biological Activity of Electrospun Nanofibers

#### 2.10.1. Antioxidant and Anti-Inflammatory Activities

The antioxidative and anti-inflammatory assays were conducted following the procedure outlined in Section 2.5.

#### 2.10.2. Cytotoxicity Assay

Human normal skin fibroblasts (Hs27 cells), purchased from the American Type Culture Collection (ATCC, Manassas, VA, USA), were incubated with extracts 1, 2, and 19 for 24 h. The MTT method was used to measure the vitality of the cells using the previously detailed methods [6].

#### 2.10.3. Wound Healing

The ability of extracts 1, 2, and 19 to repair wounds was examined on Hs27 cells using the scratch test, utilizing the previously detailed methods [6].

### 2.11. Statistical Analysis

The statistical analysis was performed using Statistica 13.3. The Shapiro–Wilk test was used to determine whether the results were normal. The ANOVA test was used to compare the mean values, and post hoc Tukey’s range test was used for multiple comparisons. At *p* < 0.05, differences across groups were deemed significant. Principal component analysis (PCA) was utilized to assess correlations using PQStat Software version 1.8.4.142 (2022).

## 3. Results and Discussion

### 3.1. Optimization of the Orange Peel Extraction Process and Characterization of Biological Activity

To obtain the most valuable extract of pomegranate peel, the Design of Experiments method was employed. Previous studies have investigated the effects of using various solvents for extraction [34,35,36]. This study chose methanol as a suitable, safe, and relatively low-cost solvent. A factorial plan was designed based on three input factors: the concentration of methanol (*v*/*v*), the temperature of the process, and extraction time. The selection of these factors was deemed appropriate due to the diversity of phytochemicals present in the plant material, the extraction of which may depend on both temperature and alcohol concentration [37].

Four quality aspects were assessed to determine the most optimal parameters: the rutin and kaempferol content, total polyphenol content, antioxidant properties, and anti-inflammatory properties (Table 3).

All nine extracts’ rutin and kaempferol contents could be found using the linearity equation of reference substances (Table 3); however, only the rutin content was used for the DoE model. The amount of methanol in the extraction mixture and the extraction temperature are two statistically significant variables influencing the rutin content, according to the Pareto diagram (Figure S1, Supplementary Materials). Additionally, both impacts are favorable because the rutin concentration rises as the temperature rises and the proportion of methanol in the extraction mixture increases. Each extract’s total polyphenol content (TPC) was ascertained in addition to its rutin content (Table 3). However, a statistical analysis of the Pareto diagram (Figure S2, Supplementary Materials) reveals that the methanol percentage is the only statistically significant factor influencing the total phenolic content. This effect also exhibits a positive sign. In the study by Elfalleh et al., the extraction effect of powdered raw material with 30% methanol at 30 °C was like the results for extracts E1–E3 obtained under similar conditions (TPC = 85.60 ± 4.87 mg GAE/g) [38]. However, the extraction time was exceeded multiple times, confirming the lack of a significant impact of prolonging the extraction process [36].

Determining the biological activity of the extracted materials is essential. The antioxidant potential (measured using three methods: DPPH, FRAP, and CUPRAC) and anti-inflammatory properties (measured by inhibition of the hyaluronidase enzyme) were assessed to assess this. Table 3 displays all the results. The percentage of methanol in the extraction mixture is a statistically significant factor affecting antioxidant activity, measured by the DPPH method (Figure S3, Supplementary Materials). At the same time, temperature was critical for anti-inflammatory activity (Figure S4, Supplementary Materials). Remarkably, both effects showed negative signs; antioxidant and anti-inflammatory activity declined (represented by an increase in IC_50_) as methanol concentration and temperature increased. Chukwuma et al. described the effects of using three different substances (water, ethanol, and acetone) to extract freshly dried and powdered raw material. The DPPH radical and FRAP assay analysis indicated that aqueous extracts exhibited better antioxidant properties than ethanol extracts. The extraction time of 24 h does not seem to be a factor affecting this aspect; however, using concentrated solvents may be crucial [35].

On the other hand, Benchagra et al. describe an analysis of an extract obtained by extraction with 70% methanol with the addition of acetic acid, using the DPPH radical to assess antioxidant activity. The obtained effect (IC_50_ = 12.49 ± 0.60 µg/mL) is slightly lower than that obtained in the present study (IC_50_ = 10.56 ± 0.17), suggesting a lack of positive effect from adding acetic acid to the extraction mixture. Other conditions do not seem to cause the lower result (extraction time 48 h, use of an ultrasonic bath) [34].

The technical features of the extraction process that yielded the extract with the best attributes and the highest activity could be determined based on test findings and statistical analysis. It was not evident from the examined cases that the extraction time was a crucial factor. Thus, it was possible to predict the model and identify the optimal parameters of the pomegranate peel extraction process based on the utility contour profiles model, which included all measured outputs (Figure S5, Supplementary Materials). These parameters were 70% methanol in the extraction mixture, 70 °C as the temperature, and three cycles every 30 min (a statistically insignificant parameter). The Design of Experiments (DOE) approach effectively enabled the design of the experiment to be carried out in a manner that allowed for the evaluation of the input parameters on the process efficiency in terms of the expected biological activities. Departing from the model of changing one factor at a time (One Factor At a Time—OFAT) in favor of a qualitative approach based on Quality by Design (QbD) allowed for material savings, including reduced consumption of reagents, electrical energy, decreased equipment wear, as well as more efficient use of time and reduced labor costs [39].

The final extract was prepared under ideal circumstances and underwent lyophilization to produce a dry extract that was utilized in further studies. The lyophilized extract was found to have antioxidant activity, as determined with DPPH radicals (IC_50_ = 9.08 ± 0.64 µg/mL), and anti-inflammatory action, as indicated by its ability to block the hyaluronidase enzyme (IC_50_ = 2.31 ± 0.12 µg/mL). The optimized extract showed broad microbiological activity against yeast *Candida albicans*; the bacteria responsible for wound infections, *Staphylococcus aureus* and *Pseudomonas aeruginosa*; the cariogenic bacterium *Streptococcus mutans*; and the periopathogens *Porphyromonas gingivalis* and *Fusobacterium nucleatum* (Table 4; Figure S6, Supplementary Materials).

Therefore, as a whole, the extract shows a favorable biological activity profile, useful from the point of view of use in oral infections and was therefore used for further research.

### 3.2. Preparation of the Electrospun Nanofibers and Analysis of Their Functionalities

The further part of the study involved obtaining nanofibers using the optimized extract with mucoadhesive biomaterials as a fiber core, according to the compositions in Table 2.

**Figure 1:**
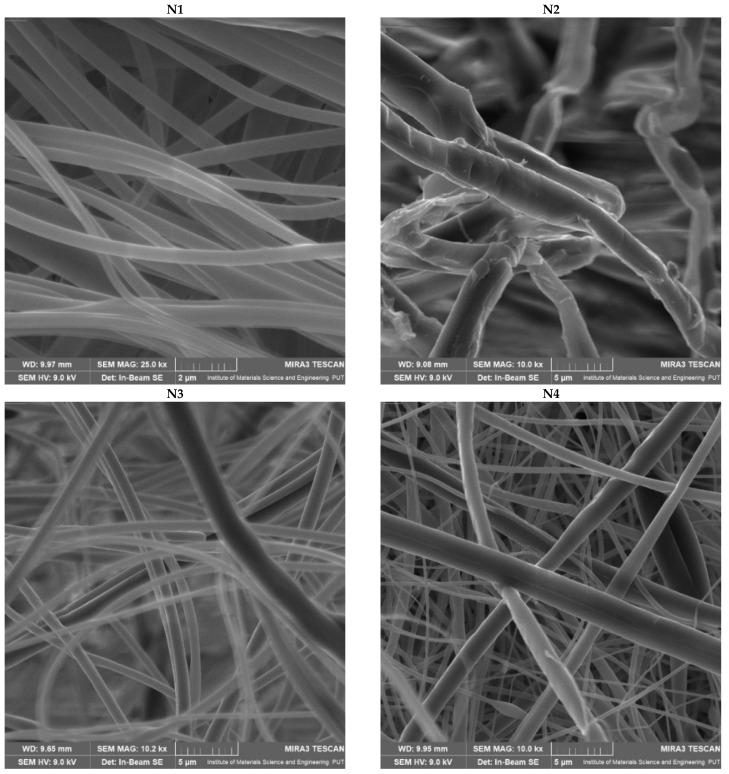

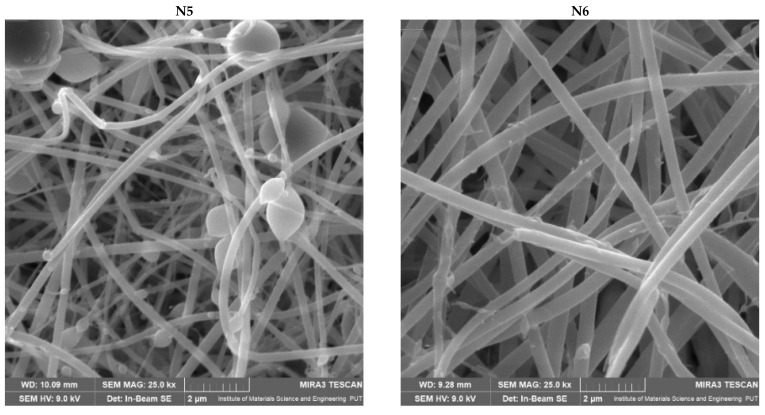
SEM images of nanofibers N1–N6.

Figure 1 shows SEM images of nanofibers N1–N6, which confirmed that the production of nanofibers went well, and good-looking nanofibers were obtained.

Based on the SEM images (Figure 1), the average diameter of the obtained nanofibers was measured. Those values were compared with the production efficiency of the electrospinning process (Table 5).

PVP–based nanofibers (N5 and N6) are characterized by excellent uniformity of fiber size and the smallest fiber diameters, which increase with an increase in PCL concentration. However, these nanofibers have beads in their structure. Interesting behavior can be observed for PCL–based nanofibers, where the polymer base has the smoothest structure (N1). At the same time, adding the extract increases the size of the nanofibers by more than four times, also causing disturbances in the smooth structure of the nanofibers (N2). A significant impact of the material used to produce nanofibers on the efficiency of the process was noticed; the highest efficiency was observed for PVP–based nanofibers (N5), and the lowest for PCL–based nanofibers (N1). Each time the extract is added, the production efficiency is reduced.

Wang et al. indicated that the poor spinnability of PVP results in a large fiber diameter, and the addition of PCL increases the spinnability of the solution, leading to finer fibers [40]. In turn, Varsei et al. found that the PVP concentration is the dominant factor (compared to the PCL concentration and other electrospinning parameters) in the morphology of nanofibers; too low or too high of a PVP concentration causes a bead-like structure [41]. Therefore, it is not the PCL concentration itself that is important; it is the ratio of PCL and PVP in the electrospun mixture. This would confirm that the smoothest structure was obtained for nanofibers containing PCL/PVP in a 1:1 ratio (nanofibers N2 and N4), which agrees with the report by Wang et al., who found that the average diameter of PCL/PVP nanofibers in a mass ratio of 1:1 was smaller and more uniform than that of pure PCL or PVP nanofibers [42].

**Figure 2:**
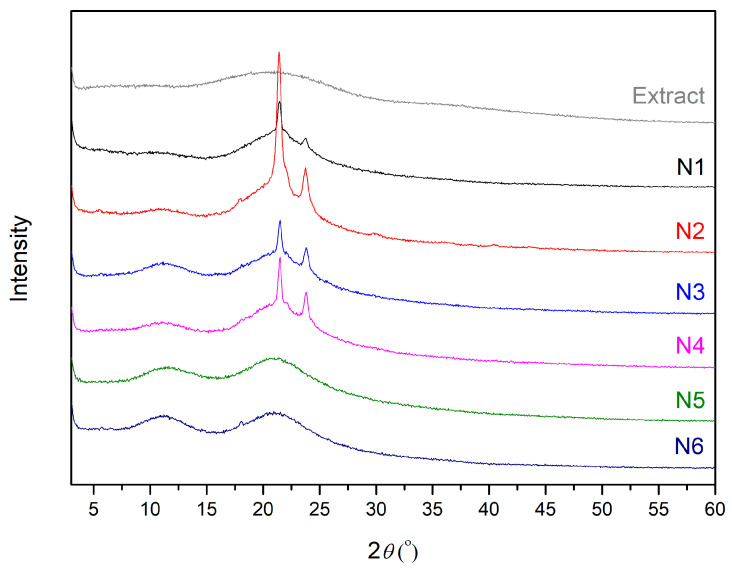
XRPD diffractograms for extract and nanofibers N1–N6.

Phase analysis by X–ray powder diffraction was performed to study the structural form of starting materials, and nanofibers N1–N6 were produced (Figure 2). When analyzing the diffractogram of lyophilized extract, its amorphous nature is visible. The two broad peaks that the raw PVP displays at about 11° and 21° indicate the amorphous nature of PVP [43]. Semi-crystalline PCL in its raw form has strong intensity peaks at 21.25° and 23.7° [44]. These peaks are visible in the analyzed nanofibers (N1–N4) made from PCL as one of the components of the prepared products. There is a prominent difference in the intensity of peaks specific for PCL between N1 and N2. N1 is combined with plant extract, and the peak is significantly lower than in N2 without plant additive. The differences between N3 and N4 are much slighter, but on the other hand, the amount of PCL is less than that in the previous pair. No differences between N5 and N6 can be observed, indicating good complexation between the nanofiber components.

The IR-ATR spectrum of the extract showed prominent absorption bands at 776, 815, 876, 1010, 1033, 1180, 1226, 1295, 1323, 1444, 1604, 1718, 2941, and 3334 cm^−1^. The IR-ATR spectrum of PVP revealed pronounced regions of absorption at 1167 cm^−1^ (C–C=O), 1229 cm^−1^ (lactone structure), 1283 cm^−1^ (C–N stretching vibrations), 1371 cm^−1^ (–CH deformation vibrations), 1420 cm^−1^ (CH_2_ wagging), 1458 cm^−1^ (CH_2_ bending vibrations), 1665 cm^−1^ (C=O), and 2951 cm^−1^ (C–H stretching vibrations), while those of PCL were at 733 cm^−1^ (C–H out of plane bending vibration), 1167 cm^−1^ (–C–O–C– symmetric stretching), 1238 cm^−1^ (C–O–C asymmetric stretching), 1294 cm^−1^ (C–O and C–C bands), 1364 cm^−1^ (stretching of OH group), 1472 cm^−1^ (stretching of CH_2_ group), 1722 cm^−1^ (–C=O stretching vibrations of the ester carbonyl group), 2866 cm^−1^ (symmetric stretching of CH_2_ group), and 2945 cm^−1^ (asymmetric stretching of CH_2_ group) [31].

**Figure 3:**
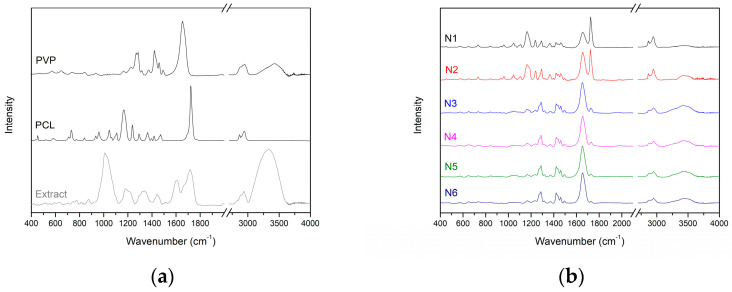
IR-ATR spectra for PVP, PCL, extract (**a**), and nanofibers N1–N6 (**b**).

The success of combined polymer spinning In combination with the extract has been demonstrated for all nanofibers. The spectra of individual nanofibers N1–N6 showed characteristic peaks for individual components: PCL for N1–N2, PCL/PVP for N3–N4, and PVP for N5–N6 (Figure 3). Moreover, there were no new, noticeable peaks in the nanofibers N1–N6. This means that the IR spectra of the nanofibers show physical interactions between PCL and PVP and the extract rather than the formation of new chemical bonds.

**Figure 4:**
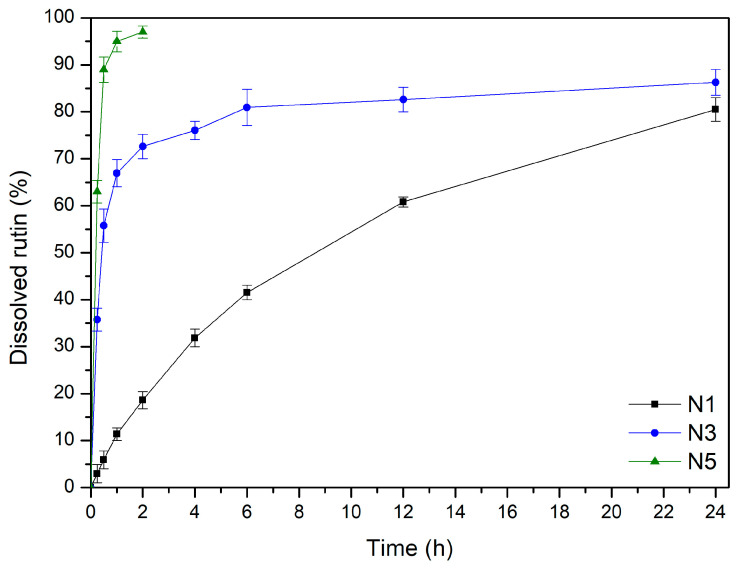
Dissolution profiles of rutin from nanofibers N1, N3, and N5.

The release of the active component from the nanofibers is a critical factor significantly affecting the product’s efficacy (Figure 4). The rutin release profiles from PCL–based, PCL/PVP–based, and PVP–based nanofibers differ considerably. Within the first 30 min, an instantaneous burst release of rutin was seen in the case of N5 nanofibers (PVP–based nanofibers). This phenomenon can be explained by using a highly hydrophilic and quickly water-soluble polymer, PVP, as well as the nanofiber structure itself, which provides a large contact surface of the substance with the acceptor fluid [28,45]. Moreover, in comparison to crystalline and semicrystalline systems, amorphous solid dispersions containing PVP show a higher apparent drug solubility and a faster rate of dissolution in aqueous solution [46]. Additionally, PVP polymer prevents recrystallization. The lack of hydrogen donor groups also affects the charge distribution of the molecule and, consequently, the crystal packing, which may explain the weak crystal structure and effective stability of the amorphous forms [47]. Higuchi kinetics, which postulates that rutin is released via diffusion across dispersed vesicles, is the most plausible release mechanism. The second likely explanation is Korsmeyer–Peppas kinetics with n over 1.0, implying non-Fickian transport (Table S1, Supplementary Material).

A prolonged release profile of rutin was noted in the cases of nanofibers N3, which were based on PCL/PVP mixture. By altering the ratio of PCL to PVP in the fibrous matrix, the hydrophilicity of PCL/PVP nanofibers can be regulated to allow sustained substance release, as PCL is a very hydrophobic polymer and PVP is a highly hydrophilic polymer [48]. Due to the rapid release of an amorphous substance on the surface of the nanofibers, the rutin release curves from nanofibers N3 may be separated into two phases: the burst release phase and the last slow-release phase. The second phase occurs according to diffusion and has the kinetic characteristics of the Higuchi model (Table S1, Supplementary Material).

Finally, the release of rutin from N1 or PCL–based nanofibers was characterized by a typical appearance for controlled release according to zero-order kinetics [49]. Thus far, the literature has reported that a modification in the fiber structure causes the release profile to slow down. Because the ester groups of the framework separate from the buffer penetration, the fibrous structures are broken, and droplet-shaped particles form in their place. Furthermore, the surface erosion of the PCL matrix causes the fibers’ surface to become rough. According to this finding, the breakdown process begins at the PCL fibers’ surface and moves inside the particles [50,51]. This may indicate re-crystallization of the guest polymer [52].

**Figure 5:**
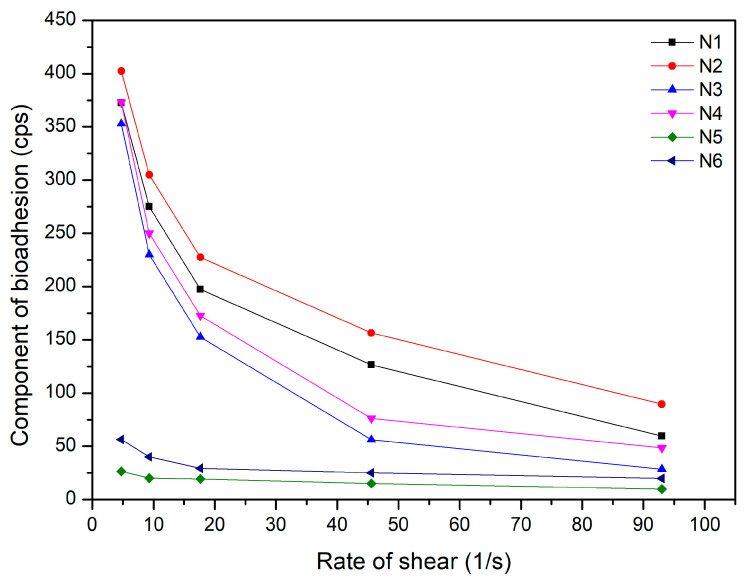
Mucoadhesive properties of nanofibers N1–N6.

To ensure prolonged release of the active substance, it is necessary to provide a long residence time for the form at the application site; therefore, such a form must have mucoadhesive properties. Thus, in this case, the mucoadhesive properties of the obtained N1–N6 nanofibers were assessed (Figure 5). The highest mucoadhesive properties were observed for the PCL–based nanofibers (N1–N2) and decreased with a decrease in the PCL content in the composition. It was also noticed that adding the extract reduced the mucoadhesive property of the entire system. As was noticed before, PVP has a more hydrophilic structure than PCL, which can result in numerous hydrogen bonds with the mucus layer. The data indicate that PCL could present hydrophobic interactions with mucus, which explains the good ability to adhere to the mucosa with a pH of around 6.8 [53].

Finally, the biological properties, i.e., the antioxidant and anti-inflammatory activities of the nanofibers, were assessed (Table 6).

Activities decreased compared to the initial extract. PVP–based (N5) nanofibers showed the most excellent activity of the produced nanofibers, related to their best solubility in water, manifested in the fastest achievement of the maximum release of the extract’s active compounds, mainly responsible for the biological action of the nanofibers. Consistently, the lowest activity was determined for PCL–based (N1) nanofibers, which is related to the limited solubility of the nanofiber base itself, and changes in the structure of nanofibers, described above.

**Figure 6:**
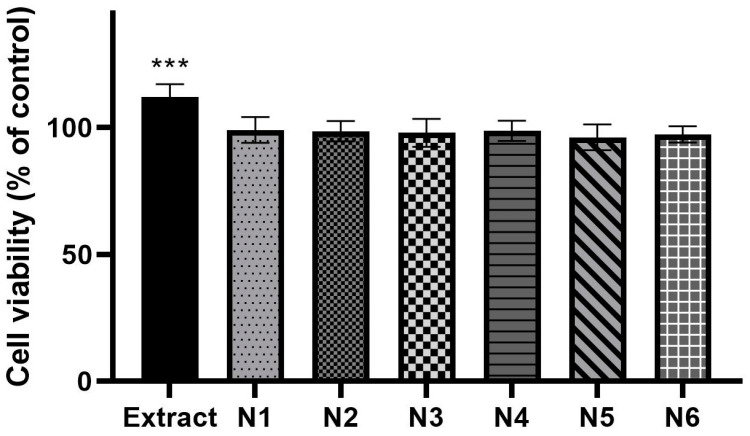
Viability of human normal skin fibroblasts (Hs27 cells) exposed to 100 µg/mL of the extract and nanofibers N1–N6 for 24 h. ANOVA statistically analyzed the results of the MTT assay with a post hoc Dunnett’s test. Statistical significance (vs control cells) was designated as *** when *p* < 0.001.

The cytotoxicity of the human normal skin fibroblasts (Hs27 cells) line was assessed using the MTT test to determine the biocompatibility of the obtained materials (Figure 6). The pure extract at a concentration of 100 µg/mL intensified the proliferation of fibroblasts, but what is important is that none of the analyzed samples disturbed (in minus) cell growth. Due to the lack of influence of the extract and nanofibers on the viability of fibroblast cells, this proves the biocompatibility of the produced material.

**Figure 7:**
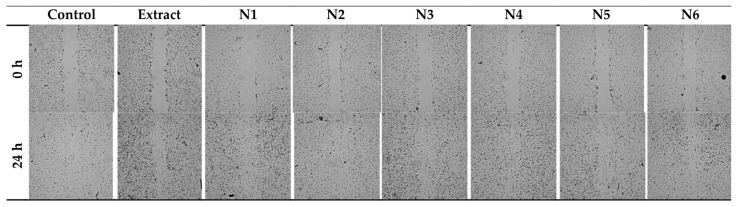
The wound-healing effect of the extract and nanofibers N1–N6 was observed on two-dimensional cultures of normal human skin fibroblasts (representative images).

**Figure 8:**
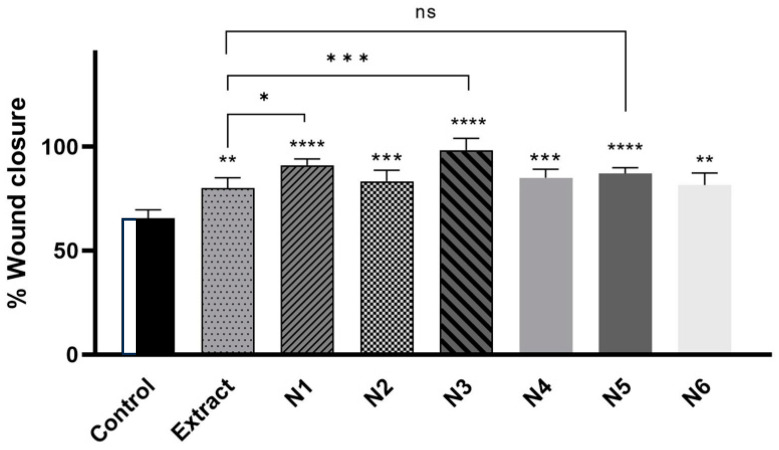
The wound-healing properties of the extract and nanofibers N1–N6 were examined on Hs27 cells after 24 h incubation. The samples were tested at a concentration of 100 µg/mL. ANOVA statistically analyzed results with a post hoc Tukey’s test. Statistical significance was designated as “*” when *p* < 0.05, “**” when *p* < 0.01, “***” when *p* < 0.001, and “****” when *p* < 0.0001 (vs. control cells or extract-treated cells); ns—not significant.

Fibroblasts are necessary for periodontal wound healing as well. These cell types are essential for the periodontal ligament, gingiva, and tooth root to renew a robust fibrillar connection [54]. Figure 7 shows the healing of the wound, and both the extract itself and the nanofibers N1, N3, N5, and their respective bases N2, N4, and N6, accelerate the healing of the scratch in a statistically significant way. The N3 system had the most potent healing effect (98.2% scratch closure versus 65.5% control closure), then N1 (91.0% vs. 65.5%), then N5 (87.1% vs. 65.5%). The extract caused the crack to heal by 80.2% (Figure 8).

The obtained nanofibers exhibited excellent biocompatibility, promoting cell proliferation, anti-inflammatory effects, and wound healing.

**Figure 9:**
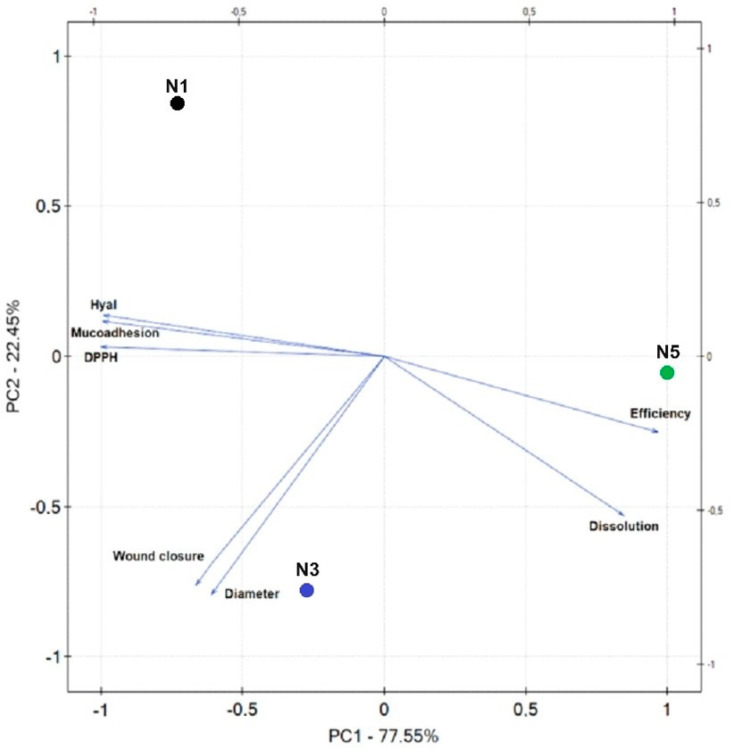
Principal component analysis (PCA) showing the factor loading plot considering the average diameter of nanofibers (=diameter), production efficiency, percentage of rutin release at 6 h (=dissolution), antioxidant activity (=DPPH), anti-inflammatory activity (=Hyal), mucoadhesive properties, and wound closure after 24 h (=wound closure); N1—nanofibers N1, N3—nanofibers N3, N5—nanofibers N5.

Figure 9 shows the results of the PCA analysis for the nanofibers’ characteristics. A statistically significant very strong correlation was indicated between the diameter of the nanofibers and wound closure (Table S2, Supplementary Materials); the larger the diameter of the nanofibers, the faster the wound healing, which indicates the importance of the material used in closing the wound. A strong correlation was demonstrated between the percentage of substances released and the antioxidant and anti-inflammatory activity, as described above, and the results from the dissolution rate of the nanofiber base; rapid dissolution of PVP and prolonged PCL resulted from the nanofiber bases’ hydrophilic–hydrophobic properties of starting materials. A very strong correlation has been demonstrated between mucoadhesive properties and biological activity; the more significant the bioadhesive component, the weaker the antioxidant and anti-inflammatory activity resulting from nanofiber bases’ hydrophilic–hydrophobic properties. The higher the mucoadhesive properties, the more hydrophobic properties, the lower the level of substance release and, consequently, the weaker the biological activity.

## 4. Conclusions

This study describes the optimization of the pomegranate peel extraction process, where it was shown that ultrasonic-assisted extraction using 70% methanol as an extractant, a temperature of 70 degrees, and conducting the process in three cycles of 60 min turns out to be the best. In the second stage, nanofibers based on PCL and/or PVP were successfully produced using electrospinning techniques and evaluated as a potential dressing material for use in the oral cavity. The developed nanofibers exhibited a pearl-free, continuous morphology. PVP–based nanofibers showed an immediate release of extract compounds, while those based on PCL showed a controlled and prolonged release over time. Finally, the potential of nanofibers to reduce the expression of inflammation and the potential of fibroblast cell proliferation were demonstrated, which indicates the possibility of accelerating wound healing. With the appropriate proportion of ingredients, PCL/PVP–based nanofibers (N3) showed the best potential. The groundbreaking strategy of fabricating electrospun nanofibers infused with pomegranate peel extract heralds a new era in pharmaceutical innovation. This pioneering approach harnesses the inherent synergy of bioactive compounds within the extract, offering unparalleled efficacy in combating oral infections. Moreover, by encapsulating these potent agents within patient-friendly nanofiber matrices, we not only optimize therapeutic delivery but also revolutionize the treatment experience, ensuring enhanced patient compliance and outcomes.

## Figures and Tables

**Table 1:** Factorial Characterization of Obtained Extracts.

Number of Extract	% Methanol Concentration (V/V)	Extraction Temperature (°C)	Extraction Time (min)
E1	30	30	30
E2	30	50	90
E3	30	70	60
E4	50	30	90
E5	50	50	60
E6	50	70	30
E7	70	30	60
E8	70	50	30
E9	70	70	90

**Table 2:** Composition of Prepared Nanofibers.

	N1	N2	N3	N4	N5	N6
PCL	1.0 g	1.0 g	0.5 g	0.5 g	-	-
PVP	1.0 g	1.0 g	1.5 g	1.5 g	2.0 g	2.0 g
Extract	0.5 g	-	0.5 g	-	0.5 g	-

**Table 3:** Content of active components, antioxidant, and anti-inflammatory activities of extracts E1–E9.

Number of Extract	Content of Active Components			Antioxidant Properties			Anti-Inflammatory Properties
	Rutin (μg/1 g Plant Material)	Kaemferol (μg/1 g Plant Material)	TPC (mg GAE/1 g Plant Material)	DPPH IC_50_ (µg/mL)	FRAP IC_0.5_ (µg/mL)	CUPRAC IC_0.5_ (µg/mL)	Inhibition of Hyaluronidase Activity IC_50_ (mg/mL)
E1	108.21 ± 0.53	0.84 ± 0.19	81.08 ± 1.40	39.64 ± 4.84	20.73 ± 1.86	22.29 ± 0.62	8.72 ± 0.28
E2	132.72 ± 1.59	0.86 ± 0.01	85.54 ± 0.18	35.02 ± 0.09	20.34 ± 1.65	22.73 ± 0.56	4.78 ± 0.11
E3	140.98 ± 0.49	1.19 ± 0.05	89.35 ± 3.76	36.09 ± 2.21	21.63 ± 1.29	21.37 ± 0.43	4.71 ± 0.05
E4	171.87 ± 5.45	5.29 ± 0.37	113.56 ± 7.39	34.47 ± 1.55	13.96 ± 0.62	16.25 ± 0.36	4.7 ± 0.10
E5	184.58 ± 3.21	5.97 ± 0.27	113.62 ± 2.82	29.33 ± 1.42	13.73 ± 0.53	14.84 ± 0.88	4.67 ± 0.20
E6	202.17 ± 17.23	4.03 ± 0.09	113.50 ± 5.71	27.97 ± 3.00	12.48 ± 0.02	14.99 ± 1.03	4.56 ± 0.26
E7	223.75 ± 3.53	10.12 ± 0.62	138.44 ± 4.68	24.40 ± 0.66	10.84 ± 1.51	12.45 ± 0.39	3.74 ± 0.98
E8	242.16 ± 15.17	9.80 ± 0.13	146.63 ± 12.07	20.74 ± 0.92	9.96 ± 0.76	10.56 ± 0.17	3.53 ± 1.02
E9	273.22 ± 4.19	7.52 ± 0.56	145.78 ± 8.22	22.98 ± 1.17	10.39 ± 0.45	11.21 ± 0.46	3.66 ± 0.90

**Table 4:** Microbiological activity of optimized pomegranate peel extract.

	MIC (mg/mL)
*Candida albicans*	6.25–12.5
*Staphylococcus aureus*	6.25–12.5
*Pseudomonas aeruginosa*	12.5
*Streptococcus mutans* ATCC 25175	6.25
*Schaalia odontolytica* (*Actinomyces odontolyticus*) ATCC 17929	3.125–6.25
*Fusobacterium nucleatum* ATCC 25586	25
*Porphyromonas gingivalis* ATCC 33277	12–25
*Prevotella intermedia* ATCC 25611	12–25

**Table 5:** Average diameter of nanofibers N1–N6, and efficiency of nanofiber production.

Number of Nanofibers	Average Diameter of Nanofibers (nm)	Nanofiber Production Efficiency (%)
N1	476.19 ± 37.90	15.79
N2	2270.21 ± 198.49	13.52
N3	1135.11 ± 73.36	47.2
N4	1162.79 ± 87.36	42.82
N5	208.19 ± 10.93	98.17
N6	376.52 ± 21.04	86.52

**Table 6:** Antioxidant and anti-inflammatory activities of nanofibers N1, N3, and N5.

Nanofibers	Antioxidant Activity	Anti-Inflammatory Activity
	IC_50_ (μg/mL)	IC_50_ (μg/mL)
N1	267.57 ± 2.94	183.73 ± 9.27
N3	244.13 ± 2.04	165.18 ± 11.19
N5	147.59 ± 0.79	120.82 ± 10.42

## Data Availability

All data supporting reported results can be found within the manuscript and Supplementary Materials.

## Supplementary Materials

The following supporting information can be downloaded at: https://www.mdpi.com/article/10.3390/ma17112558/s1, Figure S1. Statistical analysis for rutin content in extracts E1–E9: (a) Pareto plot of standardized effects for rutin content in extracts E1–E9; (b) response surface plots presenting the dependence of methanol content in the extraction mixture and extraction temperature on the rutin content in extracts for constant time of 60 min. Figure S2. Statistical analysis for total phenolic content in extracts E1–E9: (a) Pareto plot of standardized effects for total phenolic content in extracts E1–E9; (b) response surface plots presenting the dependence of methanol content in the extraction mixture and extraction temperature on the total phenolic content in extracts for constant time of 60 min. Figure S3. Statistical analysis for antioxidant activity of extracts E1–E9 measured by DPPH method: (a) Pareto plot of standardized effects for antioxidant activity; (b) response surface plots presenting the dependence of methanol content in the extraction mixture and extraction temperature on antioxidant activity of extracts for constant time of 60 min. Figure S4. Statistical analysis for anti-inflammatory activity of extracts E1–E9: (a) Pareto plot of standardized effects for anti-inflammatory activity of extracts E1–E9; (b) response surface plots presenting the dependence of methanol content in the extraction mixture and extraction temperature on anti-inflammatory activity of extracts for constant time of 60 min. Figure S5. Prediction of the optimization model for obtaining extracts based on effects with positive signs like rutin content and TPC (a) and those with negative signs like DPPH and hyaluronidase assays (b). Figure S6. Sample photos of plates with MIC testing. A—effect of pomegranate peel extract on *Streptococcus mutans* ATCC 25175, *Pseudomonas aeruginosa*, *Staphylococcus aureus*, and *Candida albicans*. After using TTC: yellow color means no growth, red color means growth of pathogens. B—effect of pomegranate peel extract (marked as GR) on *Porphyromonas gingivalis* ATCC 33277, *Prevotella intermedia* ATCC 25611, and *Schaalia odontolytica* (*Actinomyces odontolyticus*) ATCC 17929. After the use of resazurin: pink and red color means growth of periopathogens, blue and violet-brown color means no growth. Table S1. Parameters of mathematical models fitted to the chlorogenic acid release profiles of nanofibers N1, N3, and N5. Table S2. Correlation matrix.
