# Supplementary material for: Electrospun Nanofibers with Pomegranate Peel Extract as a New Concept for Treating Oral Infections
Source: Materials (Basel). 2024 May 26;17(11):2558. doi: 10.3390/ma17112558 (PMC11173823; doi:10.3390/ma17112558)
Supplement: Supplementary file 1 [file materials-17-02558-s001.zip › materials-3015273-supplementary.pdf]

materials

Supplementary Materials

Electrospun Nanofibers with Pomegranate Peel Extract as a New Concept for Treating Oral Infections

Magdalena Paczkowska-Walendowska [1]*, Miłosz Ignacyk [1], Andrzej Miklaszewski [2], Tomasz Plech [3], Tomasz M. Karpiński [4], Jakub Kwiatek [5], Ewelina Swora-Cwynar [6], Michał Walendowski [7] and Judyta Cielecka-Piontek [1]

1 Department of Pharmacognosy and Biomaterials, Poznan University of Medical Sciences, Rokietnicka 3, 60-806 Poznan, Poland; m.ignacyk99@gmail.com (M.I.); jpiontek@ump.edu.pl (J.C.-P.)
2 Faculty of Materials Engineering and Technical Physics, Institute of Materials Science and Engineering, Poznan University of Technology, 60-965 Poznan, Poland; andrzej.miklaszewski@put.poznan.pl
3 Department of Pharmacology, Medical University of Lublin, Radziwillowska 11, 20-080 Lublin, Poland; tomasz.plech@umlub.pl
4 Department of Medical Microbiology, Medical Faculty, Poznan University of Medical Sciences, Rokietnicka 10, 60-806 Poznan, Poland; tkarpin@ump.edu.pl
5 Kwiatek Dental Clinic Sp. z o.o., Kordeckiego 22, 60-144 Poznan, Poland; jakubkwiatek@klinikakwiatek.pl 6 Department of Pharmacology and Phytochemistry, Institute of Natural Fibres and Medicinal Plants—National Research Institute, Wojska Polskiego 71b, 60-630 Poznan, Poland; eswora@ump.edu.pl
7 Science-Bridge Sp. z o.o., Chociszewskiego 24/8, 60-258 Poznan, Poland; michalwalendowskipoczta@gmail.com
* Correspondence: mpaczkowska@ump.edu.pl

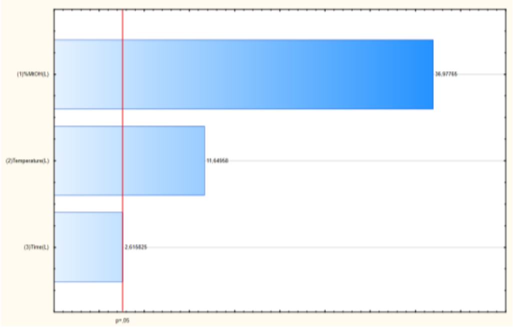

(a)

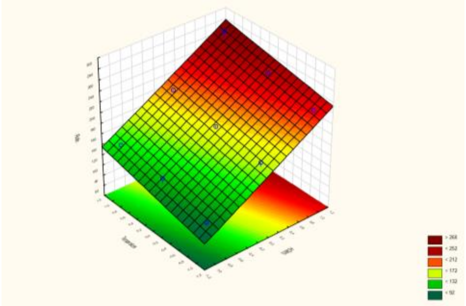

(b)

Figure S1. Statistical analysis for rutin content in extracts E1-E9: (a) Pareto plot of standardized effects for rutin content in extracts E1-E9; (b) Response surface plots presenting the dependence of methanol content in the extraction mixture and extraction temperature on the rutin content in extracts for constant time of 60 minutes.

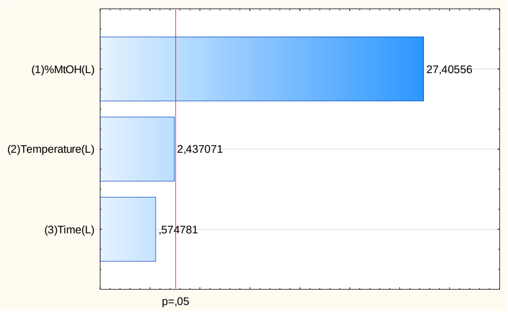

(a)

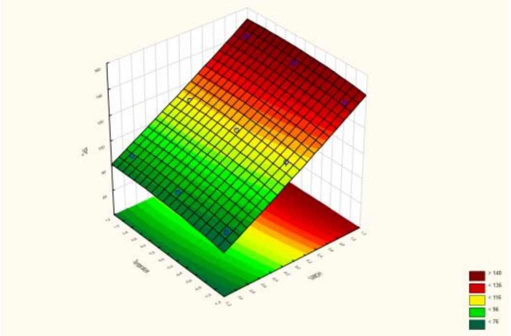

(b)

Figure S2. Statistical analysis for total phenolic content in extracts E1-E9: (a) Pareto plot of standardized effects for total phenolic content in extracts E1-E9; (b) Response surface plots presenting the

dependence of methanol content in the extraction mixture and extraction temperature on the total phenolic content in extracts for constant time of 60 minutes.

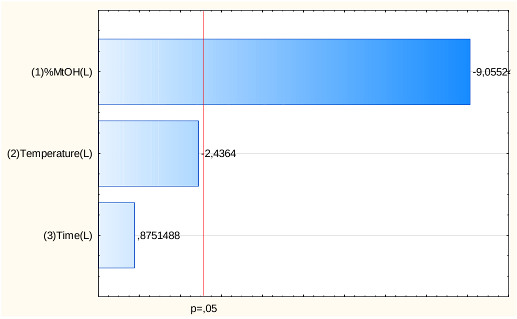

(a)

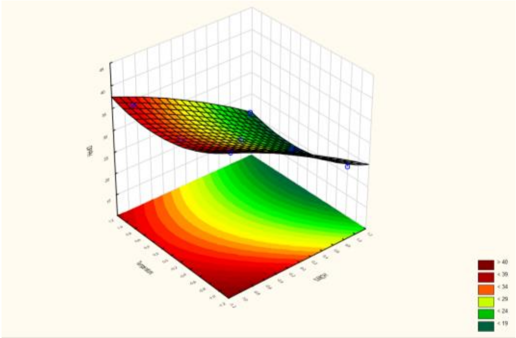

(b)

Figure S3. Statistical analysis for antioxidant activity of extracts E1-E9 measured by DPPH method: (a) Pareto plot of standardized effects for antioxidant activity; (b) Response surface plots presenting the dependence of methanol content in the extraction mixture and extraction temperature on antioxidant activity of extracts for constant time of 60 minutes.

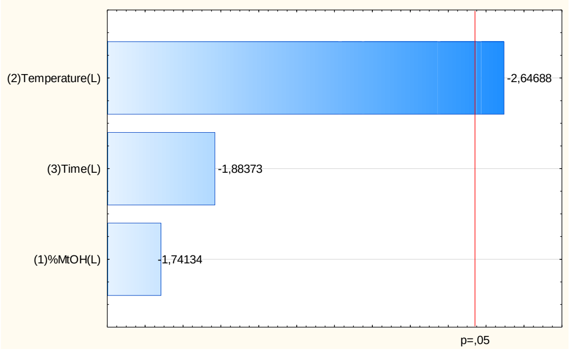

(a)

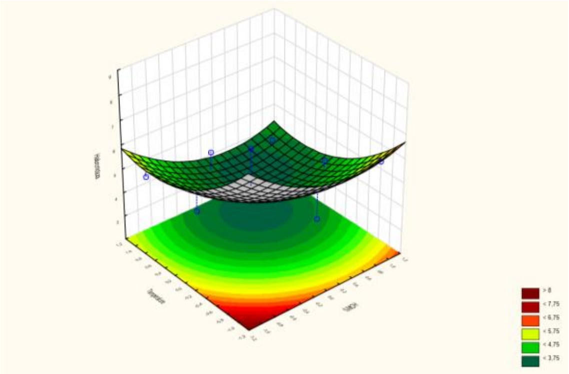

(b)

Figure S4. Statistical analysis for anti-inflammatory activity of extracts E1-E9: (a) Pareto plot of standardized effects for anti-inflammatory activity of extracts E1-E9; (b) Response surface plots presenting the dependence of methanol content in the extraction mixture and extraction temperature on anti-inflammatory activity of extracts for constant time of 60 minutes.

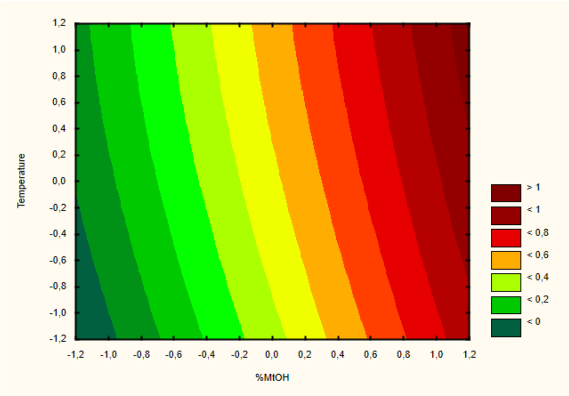

(a)

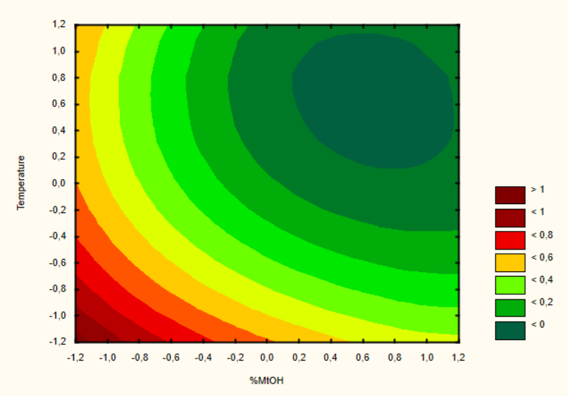

(b)

Figure S5. Prediction of the optimization model for obtaining extracts based on effect with positive sign like rutin content and TPC (a) and those with negative sing like DPPH and hyaluronidase assays (b).

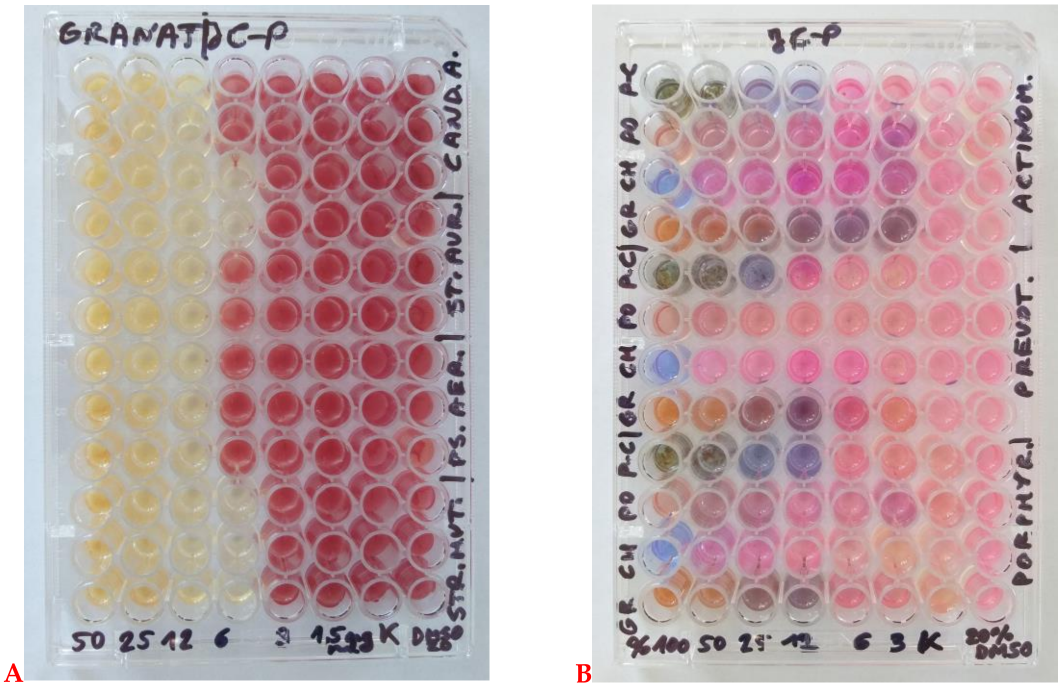

A B

Figure S6. Sample photos of plates with MIC testing. A - effect of pomegranate peel extract on *Streptococcus mutans* ATCC 25175, *Pseudomonas aeruginosa*, *Staphylococcus aureus* and *Candida albicans*. After using TTC: yellow color means no growth, red color means growth of pathogens. B - effect of pomegranate peel extract (marked as GR) on *Porphyromonas gingivalis* ATCC 33277, *Prevotella intermedia* ATCC 25611 and *Schaalia odontolytica* (*Actinomyces odontolyticus*) ATCC 17929. After the use of resazurin: pink and red color means growth of periopathogens, blue and violet-brown color means no growth.

Table S1. Parameters of mathematical models fitted to the chlorogenic acid release profiles of nanofibers N1, N3 and N5.

Formulation	**Mathematical model**							
No.	**Zero-order kinetic**		**First-order kinetic**		**Higuchi kinetic**		**Korsmeyer-Peppas kinetic**	
	K	R^2	K	R^2	K	R^2	R^2	n
N1	**3.36**	**0.90**	0.14	0.53	8.66	0.88	**0.97**	**0.53**
N3	2.03	0.33	0.07	0.13	**9.98**	**0.84**	0.61	0.46
N5	36.50	0.50	1.46	0.34	**22.74**	**0.85**	**0.74**	**1.05**

the most fitting mathematical model is shown in bold.

Table S2. Correlation matrix

	Diameter	Efficiency	Dissolution in 6 hours	Antioxidant activity (DPPH)	Anti-inflammatory activity (Hyal)	Mucoadhesive properties	Wound closure in 24 hours
Diameter		-0.3877	-0.0945	0.5865	0.5032	0.5106	**0.9976**
Efficiency	-0.3877		**0.9543**	**-0.9740**	**-0.9917**	**-0.9905**	-0.4502
Dissolution in 6 hours	-0.0945	**0.9543**		**-0.8617**	**-0.9078**	**-0.9042**	-0.1628
Antioxidant activity (DPPH)	0.5865	**-0.9740**	**-0.8617**		**0.9951**	**0.9959**	0.6409
Anti-inflammatory activity (Hyal)	0.5032	**-0.9917**	**-0.9078**	**0.9951**		**0.9999**	0.5615

Mucoadhesive properties	0.5106	**-0.9905**	**-0.9042**	**0.9959**	**0.9999**		0.5686
Wound closure in 36 hours	**0.9976**	-0.4502	-0.1628	0.6409	0.5615	0.5686	

strong and very strong correlations are shown in bold
